# Gravity-Dependent Blind Nasotracheobronchial Suction for Total Lung Collapse: A Report of Two Cases

**DOI:** 10.7759/cureus.84965

**Published:** 2025-05-28

**Authors:** Johanna Blagoie, Aravind Reddy Polam, Venkata Buddharaju, Shreeya Buddaraju

**Affiliations:** 1 Internal Medicine, Weiss Memorial Hospital, Chicago, USA; 2 Pulmonary and Critical Care Medicine, Weiss Memorial Hospital, Chicago, USA; 3 Neuroscience, University of Michigan, Ann Arbor, USA

**Keywords:** acute hypoxic respiratory failure, mucus plug impaction, nasotracheal suction, nasotracheobronchial suction, pulmonary atelectasis

## Abstract

Mucus plugging can lead to significant respiratory complications, especially in elderly or debilitated patients with impaired cough reflexes. Total lung collapse may occur when a main bronchus is occluded, often requiring bronchoscopy for diagnosis and treatment. However, bronchoscopy may not be feasible or an option in patients with comfort care measures or do-not-intubate status. We present the cases of two patients with total lung collapse due to mucus plugging who were successfully managed with bedside blind nasotracheobronchial suctioning with marked clinical and radiographic improvement, thus avoiding the need for more invasive bronchoscopy procedures. These cases highlight a practical and effective bedside alternative for managing lung collapse due to airway mucus obstruction in patients who are less likely to tolerate invasive interventions requiring sedation and bronchoscopy.

## Introduction

Pulmonary atelectasis can arise from intrinsic or extrinsic compression of the airways. Intrinsic causes include mucus plugging or foreign body aspiration, while extrinsic causes may involve lymphadenopathy or malignancy [1]. Mucus plugs are composed of desquamated bronchial epithelium and secretions and are commonly seen in patients with diminished cough reflexes, such as those with stroke, dementia, or advanced lung disease. Obstruction of a peripheral bronchus causes segmental atelectasis, whereas occlusion of a main bronchus can result in total lung collapse [2].

These events are particularly common in postoperative and perioperative settings due to diaphragmatic paralysis from anesthesia, reduced mucociliary clearance, and impaired respiratory drive. Mucus plugs are also common in patients with chronic obstructive pulmonary disease (COPD), with bronchitis predominant, pneumonia, and cystic fibrosis [3]. If untreated, these mucus plugs can accumulate over time and can cause hypoxia, hypercapnia, and increased pulmonary vascular resistance, sometimes requiring invasive procedures such as bronchoscopy to clear the mucus if patients are unable to clear it with a natural cough. However, in patients for whom invasive procedures are not an option, bedside nasotracheal suction is essential to remove mucus from the airways [4]. In cases of total lung collapse, nasotracheobronchial suction of the collapsed bronchus is rarely performed and reported in the literature. We report two cases in which nasotracheobronchial suctioning was successfully used to relieve main bronchus obstruction and to re-expand collapsed lungs. We have also described this technique in the discussion with the hope that healthcare providers can learn and perform this procedure to improve oxygenation and ventilation in patients with total lung collapse due to mucus plugs related to main bronchus obstruction. 

## Case presentation

Case 1

A 93-year-old female patient with a do-not-resuscitate (DNR)/do-not-intubate (DNI) status was admitted from a skilled nursing facility due to worsening dyspnea and hypoxia. Her past medical history included dementia, epilepsy, chronic hypoxic respiratory failure (requiring 2-3 L/min nasal cannula at baseline), hypertension, and a prior pulmonary embolism on rivaroxaban. 

**Figure 1:**
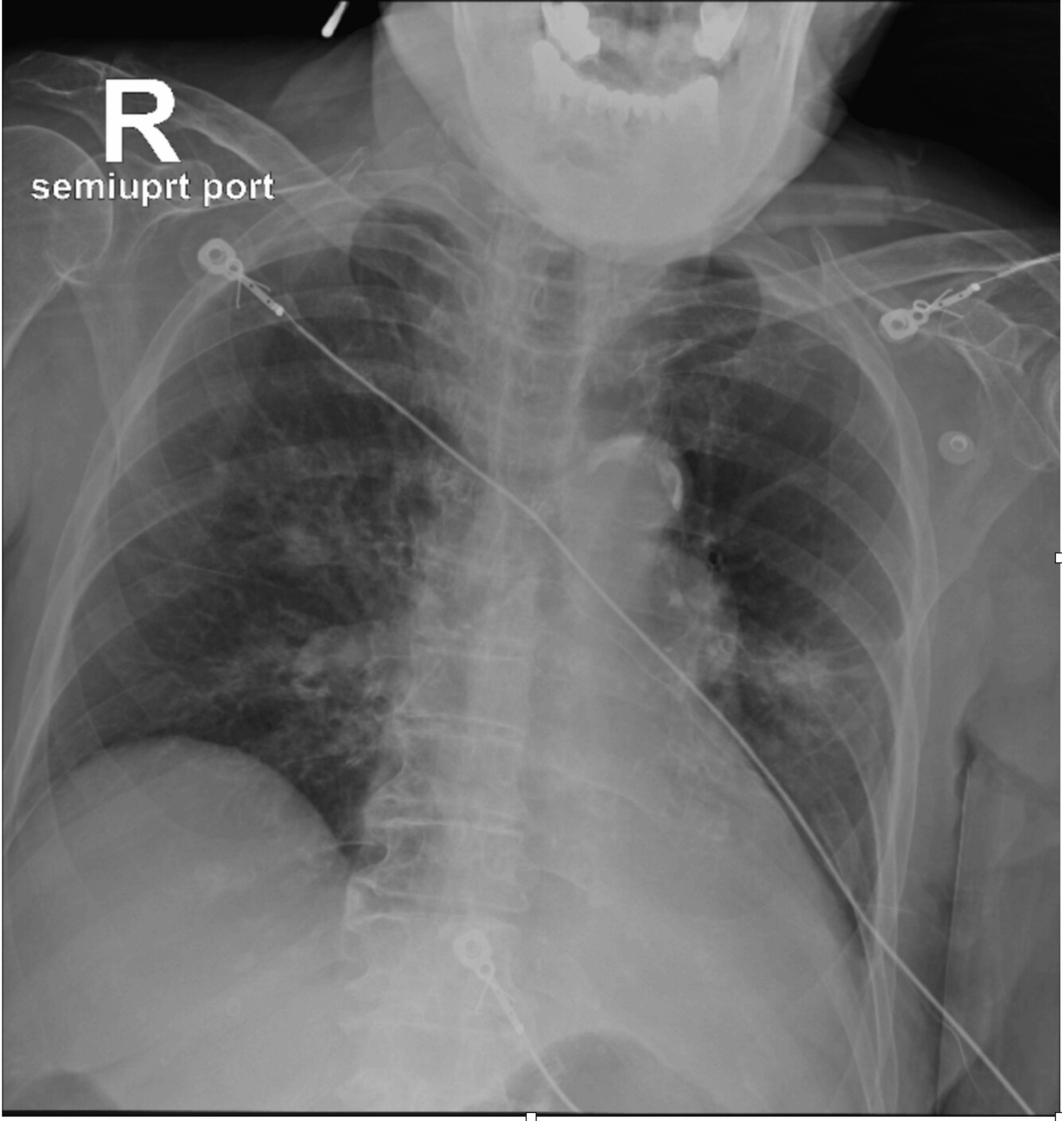
Chest X-ray at the presentation showing left lung infiltrates and consolidation.

On presentation, she was tachypneic and desaturated to 90%, requiring bilevel positive airway pressure (BiPAP). Laboratory data revealed a white blood cell count of 9.5 × 10⁹/L and lactate of 4.4 mmol/L. Chest radiograph (Figure 1 ) demonstrated left mid-lung consolidation and right perihilar patchy infiltrates, and he was diagnosed with pneumonia and started on intravenous antibiotics, fluids, glycopyrrolate, and bronchodilators with bedside airway suction as needed. 

**Figure 2:**
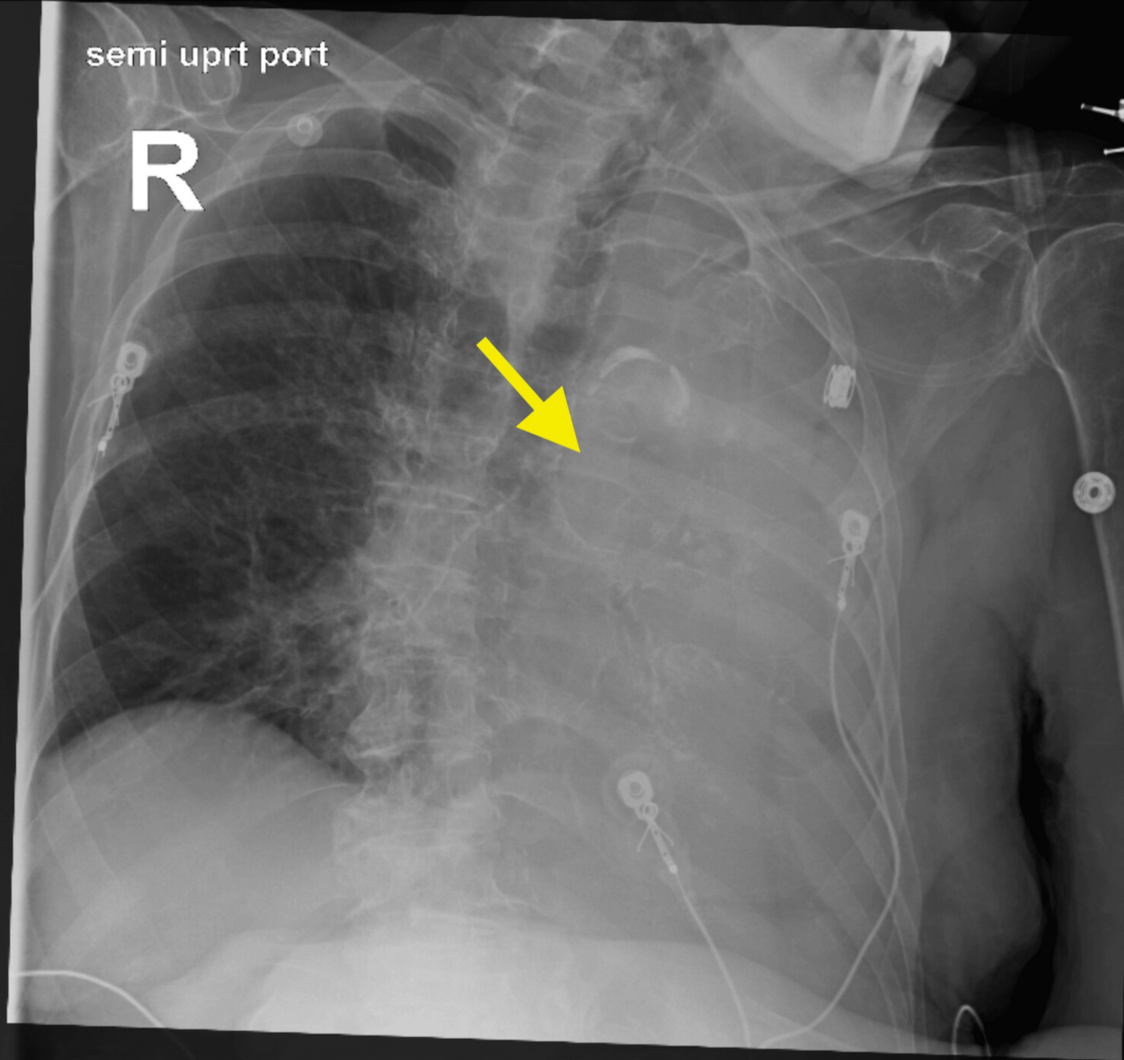
Chest X-ray, two days after admission, showing complete collapse of the left lung, with a yellow arrow pointing to the left lung hilar area.

By hospital day two, oxygen requirements increased. Chest X-ray (Figure 2) showed complete left lung collapse. 

Pulmonology was consulted, but bronchoscopy and intubation were declined by the family in accordance with the patient’s code status. Bedside nasotracheobronchial suctioning directed toward the left main bronchus was performed, and thick, tenacious, brownish-yellow secretions were removed. 

**Figure 3:**
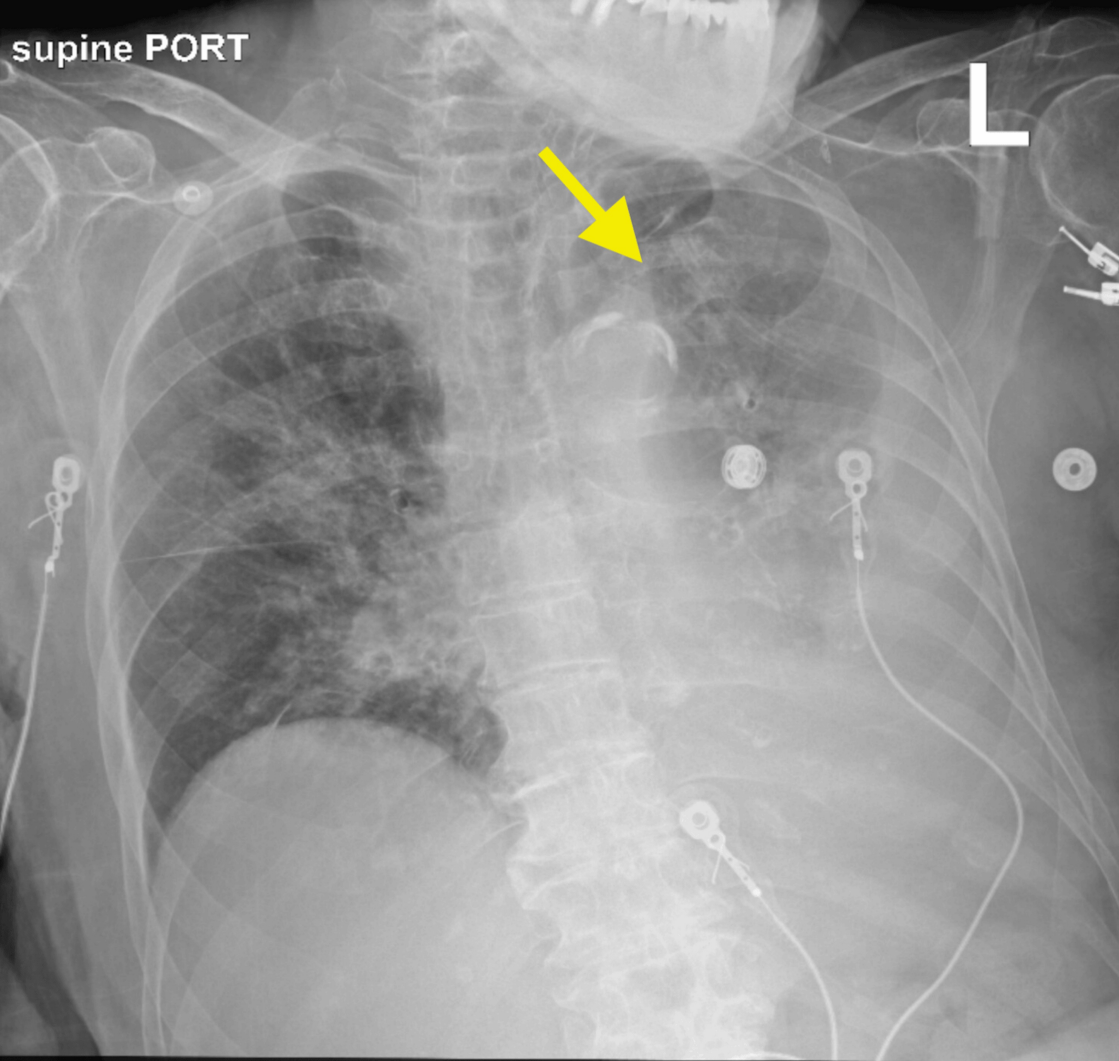
Chest X-ray, taken six hours after nasotracheobronchial suction, showing partial expansion of the left lung, with a yellow arrow pointing to the left upper lobe.

**Figure 4:**
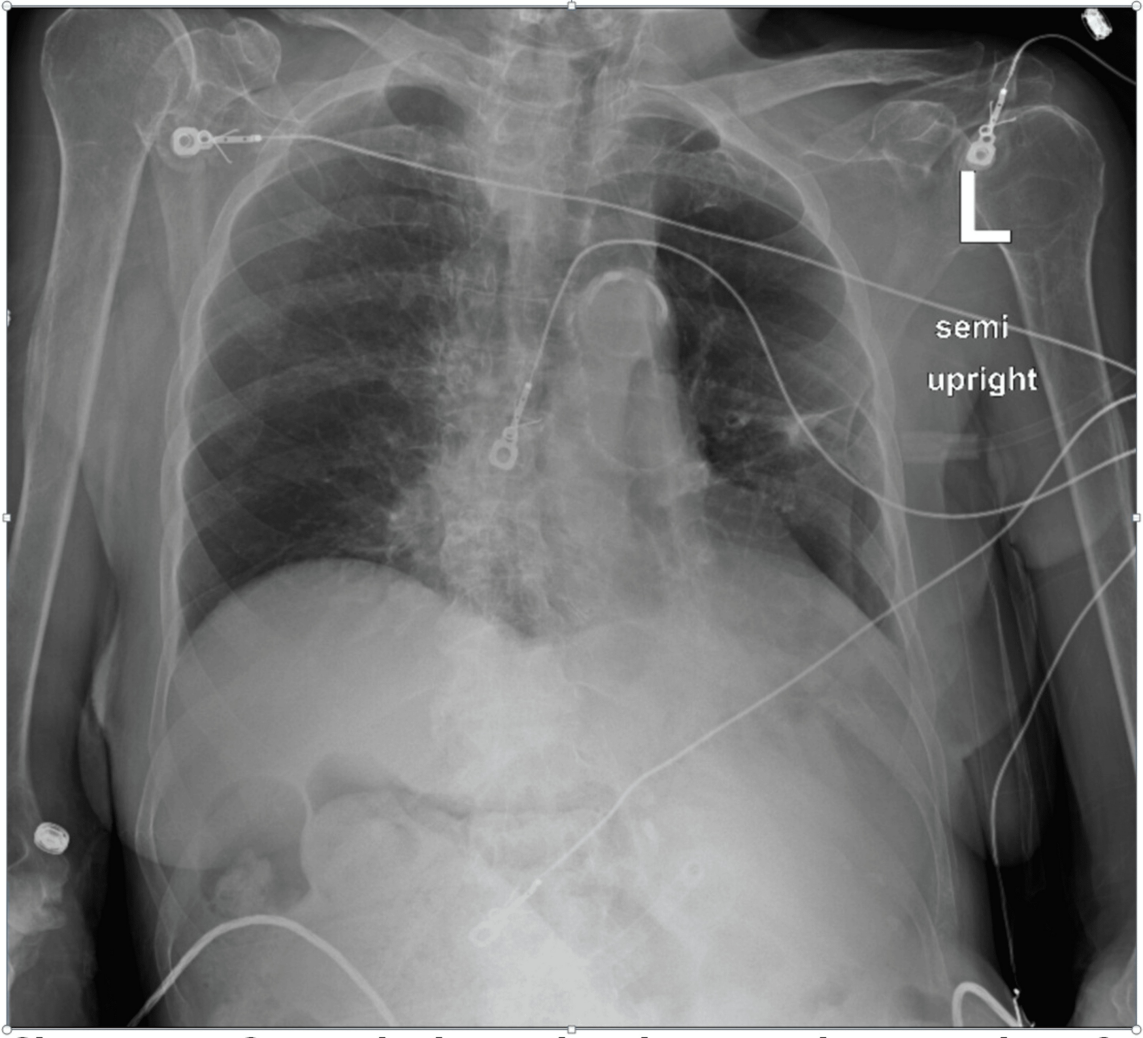
Chest X-ray two months after nasotracheobronchial suction showing complete left lung expansion.

Follow-up chest X-ray (Figure 3) six hours after nasotracheobronchial suction showed improved aeration of the left upper lobe, with residual left lower lobe atelectasis. Chest physiotherapy and pulmonary hygiene were initiated, and the patient’s oxygen saturation improved to her pre-admission baseline, and she was discharged from the hospital. Chest X-ray (Figure 4) available after two months showed complete left lung expansion. 

Follow-up chest X-ray after two months showed complete resolution of left lung collapse. 

Case 2

**Figure 5:**
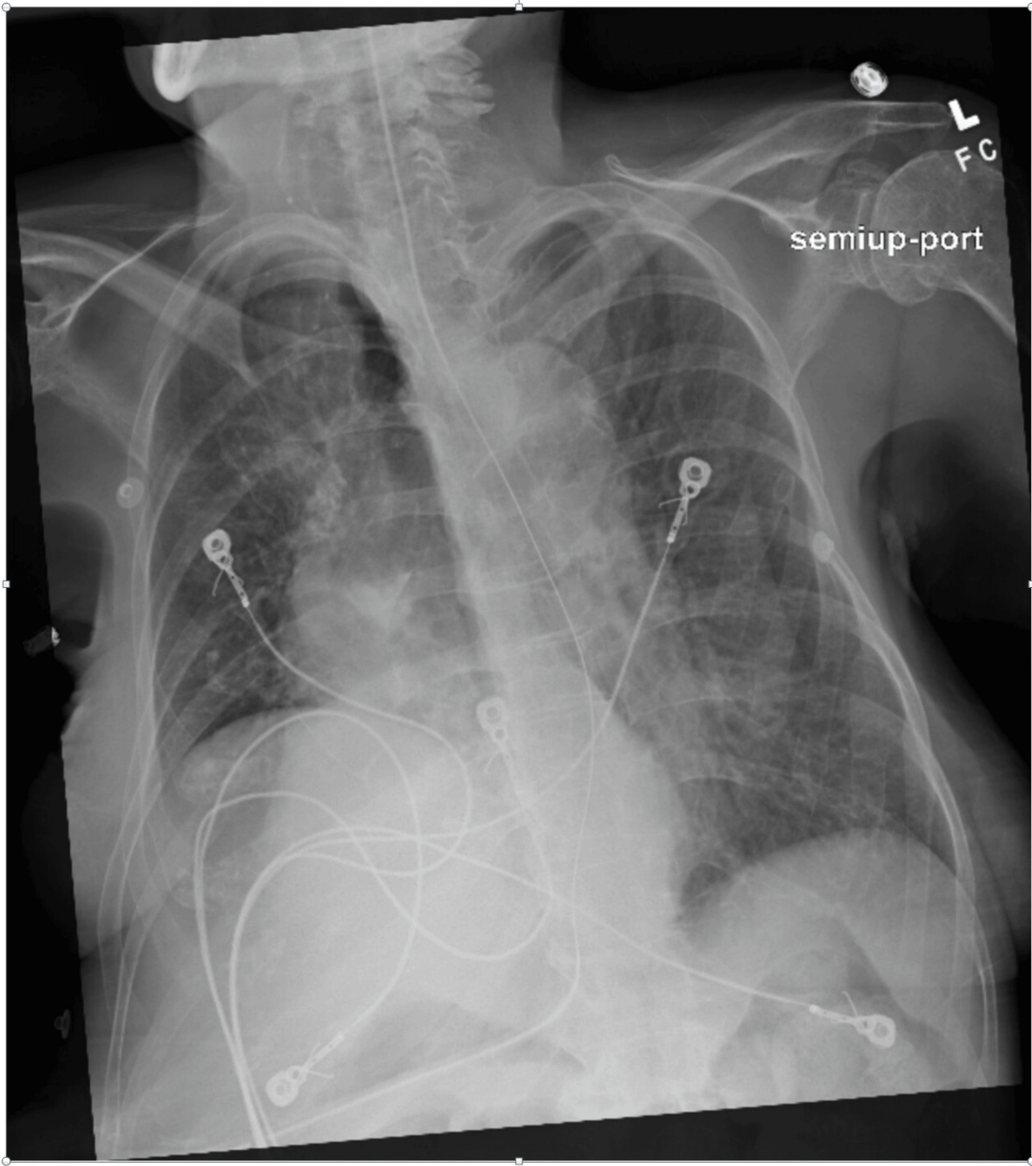
Chest X-ray at presentation showing pulmonary congestion.

A 75-year-old female patient from a skilled nursing facility presented with anemia with a past medical history of COPD on room air, diabetes mellitus, chronic anemia, hyperlipidemia, parkinsonism, Alzheimer’s dementia, schizophrenia, depression, and osteoarthritis. Physical examination revealed a grade 4/6 systolic murmur, and echocardiography confirmed severe aortic stenosis. Chest radiograph (Figure 5) at the time of admission showed pulmonary vascular congestion.

She underwent esophagogastroduodenoscopy (EGD) for evaluation of anemia, which was normal. Colonoscopy revealed a 20 cm tubulovillous adenoma requiring surgical resection. Due to her poor prognosis, severe aortic stenosis, and elevated international normalized ratio (INR) (3.3), surgery was deferred. Her code status was changed to DNR/DNI.

**Figure 6:**
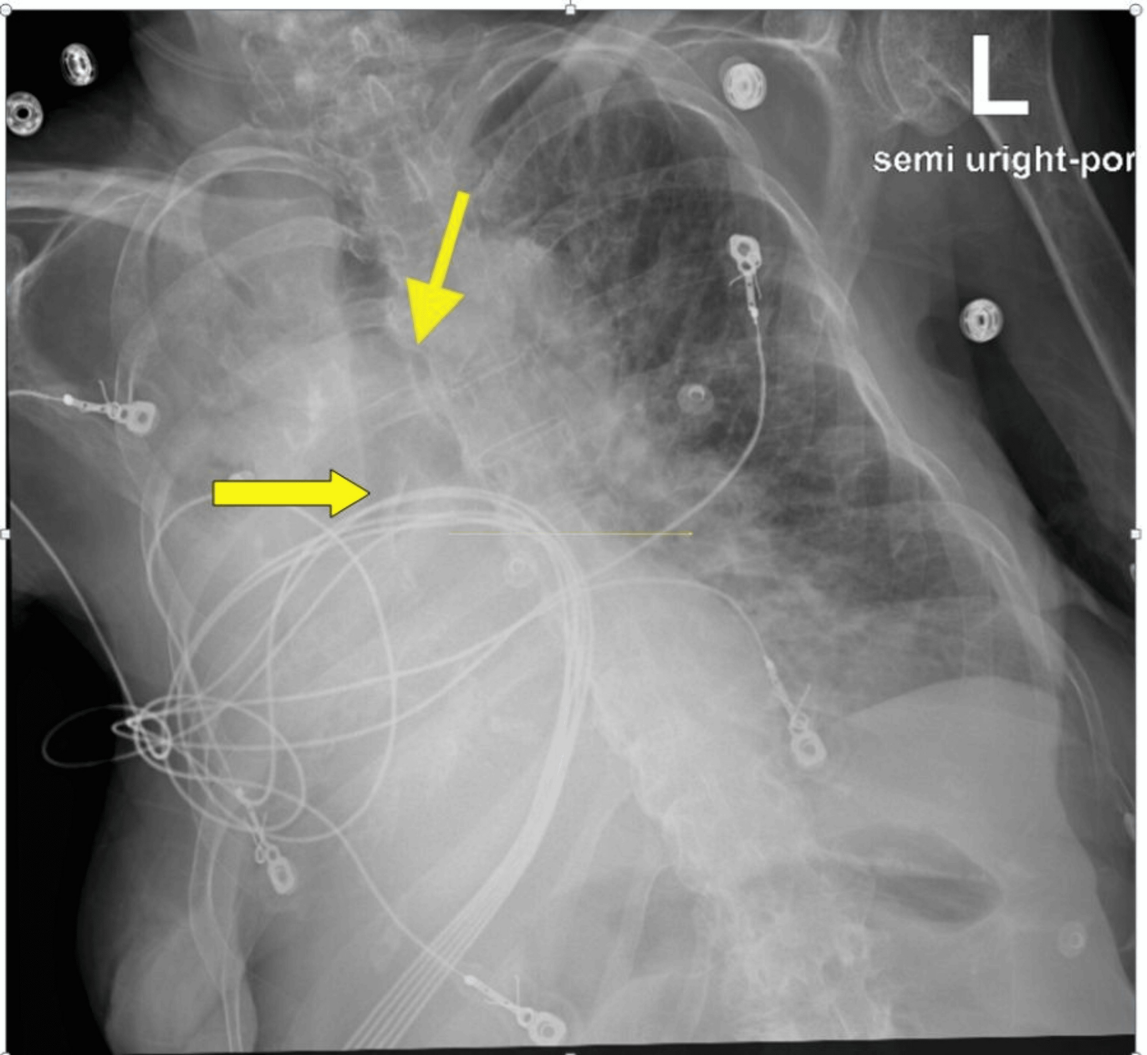
Chest X-ray showing complete collapse of the right lung, with an upper angled arrow pointing to the trachea and a horizontal lower arrow pointing to the right main bronchus, indicating cutoff of the air bronchogram.

Subsequent chest radiograph (Figure 6) during the hospital course showed complete right lung collapse, and the patient developed increased work of breathing, requiring 10 L/min of high-flow nasal cannula to maintain pulse oxygen at 97%. Arterial blood gas showed severe respiratory acidosis (pH: 7.09, partial pressure of carbon dioxide (PaCO₂): 92 mmHg, partial pressure of oxygen (PaO₂): 99 mmHg, bicarbonate (HCO₃): 27 mmol/L). She was then started on BiPAP to help improve oxygenation and ventilation. 

**Figure 7:**
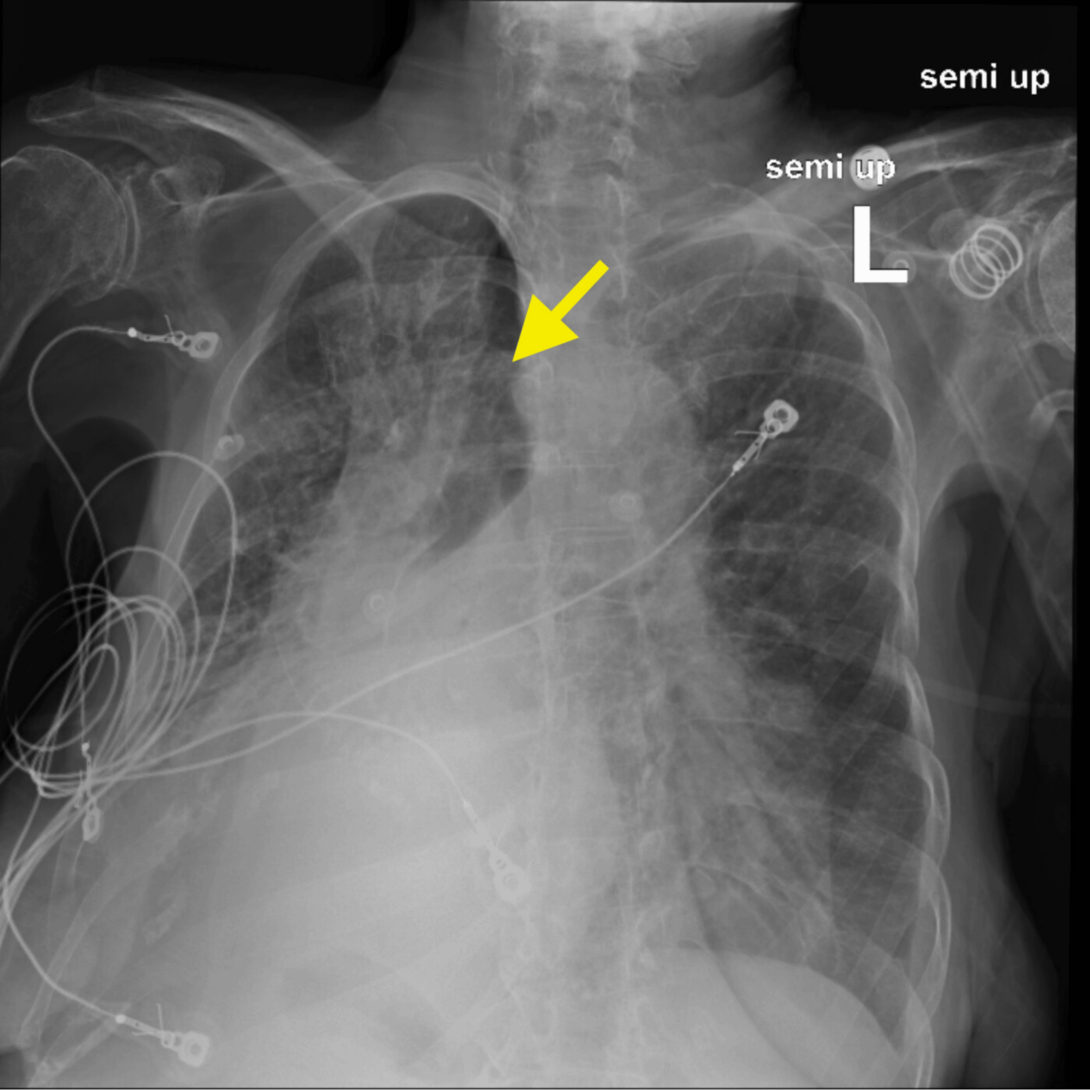
Chest X-ray, partially rotated, taken 24 hours after nasotracheobronchial suction, showing expansion of the right lung, with a yellow angled arrow pointing to the right side of the tracheobronchial tree.

**Figure 8:**
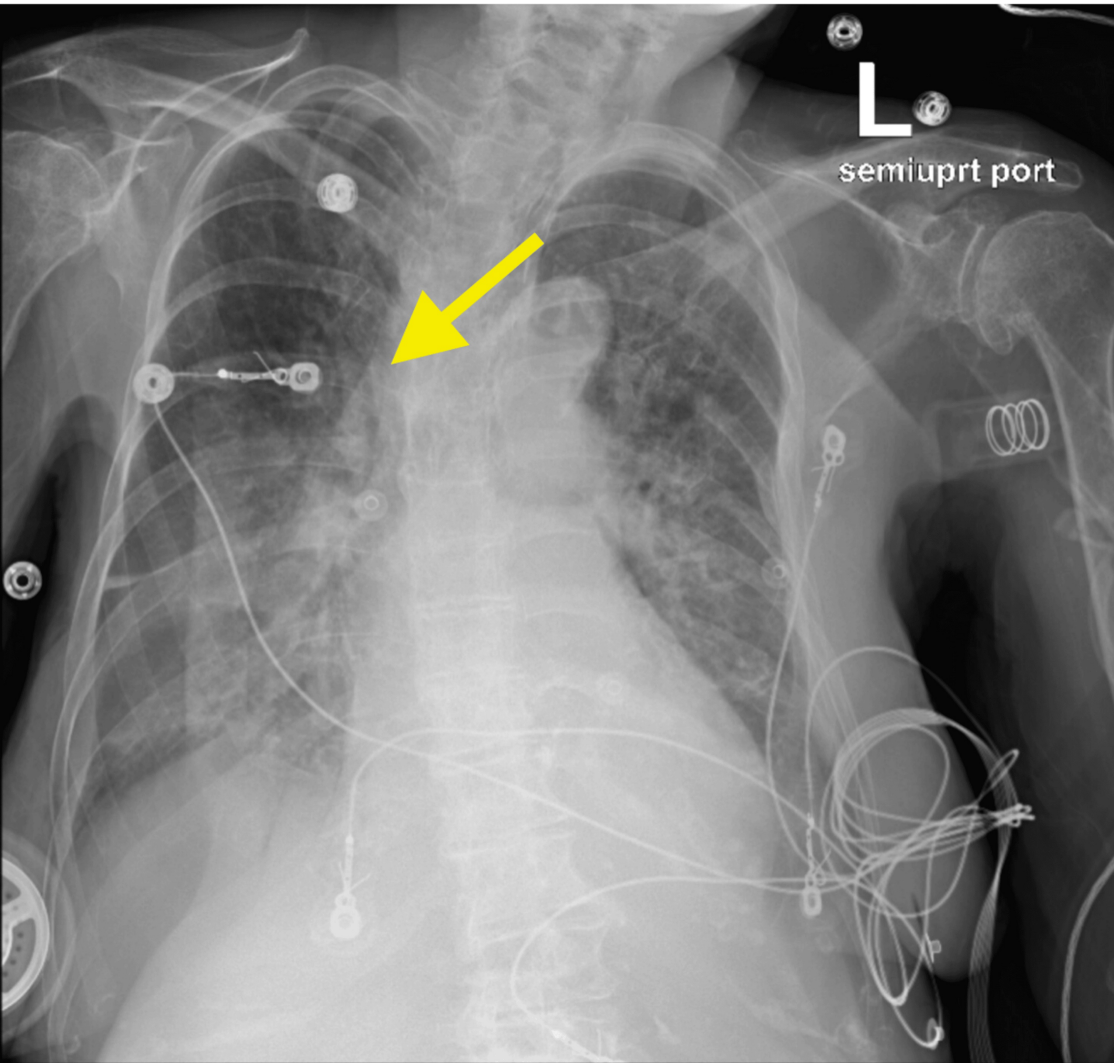
Chest X-ray taken two days after nasotracheobronchial suction, showing complete expansion of the right lung, with the yellow arrow pointing to the right lung hilar area.

Due to her DNR/DNI status, bedside gravity-assisted nasotracheobronchial suctioning directed to the right main bronchus was performed. Thick mucus plugs were removed, and a follow-up chest X-ray (Figure 7) performed after 24 hours showed a partially rotated film with re-expansion of the right lung. Chest X-ray (Figure 8) performed after two days showed complete expansion of the right lung. 

She was subsequently weaned from BiPAP and high-flow to a nasal cannula with oxygen saturations maintained above 90%.

## Discussion

**Figure 9:**
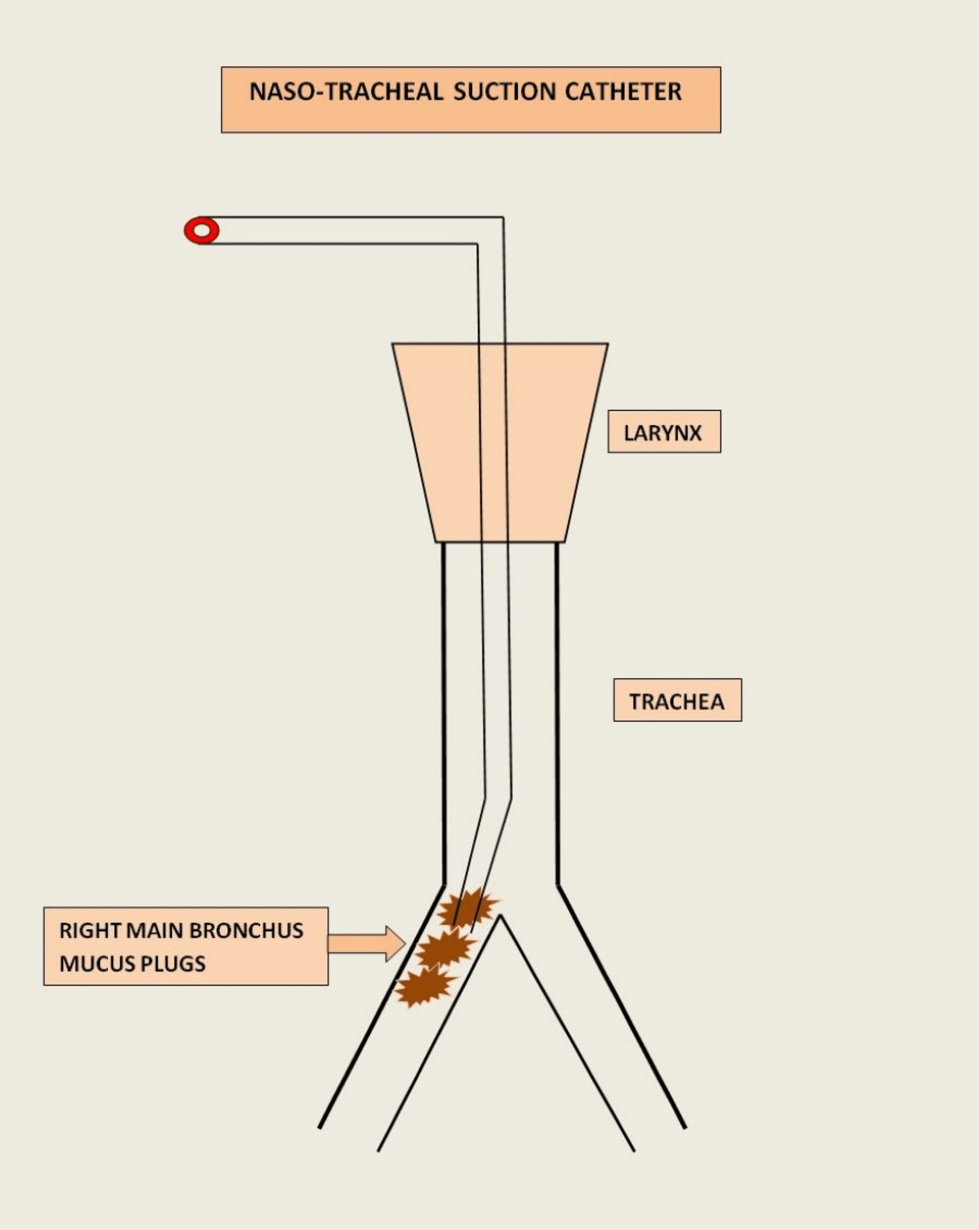
Artistic depiction of nasotracheobronchial suction This image has been used with permission from contributing author Venkata Buddharaju, MD (Pulmonary and Critical Care Medicine, Weiss Memorial Hospital, Chicago)

Figure 9 shows the technique of gravity-dependent nasotracheobronchial suction.

The patient was positioned supine with the body tilted 30 degrees toward the side of the collapsed lung. Oxygen was delivered via nasal cannula at 4-8 L/min to maintain oxygen saturation above 94%. A 12 French (4.0 mm), 40 cm nasotracheal suction catheter was lubricated and inserted into the nostril on the affected side. Using a “wait-and-advance” technique, the catheter was gently directed into the posterior pharynx and trachea, with advancement guided by patient cough and an angled trajectory toward the collapsed lung.

Suction was intermittently applied by opening and closing the suction port, with suction limited to 10-15 seconds per pass to prevent desaturation. Copious yellowish secretions were aspirated and collected in a trap for culture. Post-procedure chest radiographs showed significant improvement in lung aeration. Anatomically, the average distance from the nostril to tracheal bifurcation is 28-30 cm, allowing effective access to the main bronchi. This technique avoids the need for sedation, bronchoscopy, or advanced procedural resources.

Given the patient's goals of care to be more selective without aggressive procedures such as bronchoscopy, this procedure can be safely performed as an extension of nasotracheal suction. One needs to advance the suction catheter closer to the collapsed lung side of the main bronchus by slightly tilting the patient towards the collapsed lung, using gravity as a guide, hoping to remove the impacted mucus and expand the lung. The only guide that you are going into the vocal cords and trachea is cough, and with the wait and proceed maneuver, the suction catheter can be advanced to see if you are able to collect mucus in the suction trap and improve oxygenation. It is done blindly, as are most nasotracheal suctions performed at the bedside by respiratory therapists or nurses. The complication one would want to watch for is hypoxemia during airway suction, so the recommendation is to limit the airway suction to 10-15 seconds at a time, which is a standard practice to limit hypoxemia. The other recommendation is to monitor the patient's pulse oximetry continuously at the bedside during the suction and hold and resume airway suction based on the pulse oxygen levels to keep the oximetry above 90%.

Discussion of the cases

The lungs maintain a protective environment against pathogens through a sophisticated mechanism involving airway mucus. This mucus is primarily composed of water, with smaller quantities of mucin glycans and other peptides that help trap inhaled pathogens. Clearance of these trapped particles relies on coordinated ciliary beating and the cough reflex, which propel mucus toward the distal airways for expulsion. Disruptions in this system, such as mucus deficiency, ciliary dysfunction, excessive mucus production, or abnormally thick and sticky mucus (as seen in cystic fibrosis or smokers), can impair clearance and contribute to the development of airway diseases [5]. Mucus plugs are composed of desquamated bronchial epithelial cells and secretions that are normally cleared as part of the mucociliary clearance system. Excess mucus production is commonly observed in conditions such as pneumonia, bronchitis, and hypersecretory states like chronic bronchitis and COPD. In patients with impaired cough reflex, such as those with stroke, decreased consciousness, or neurodegenerative conditions such as Parkinson's disease, mucus clearance is significantly reduced, leading to the accumulation of thick secretions that may obstruct the airways. When mucus becomes lodged in the bronchial tree, it can result in distal lung collapse. Over time, this accumulation may cause partial or complete obstruction of a segmental, lobar, or even main bronchus, resulting in varying degrees of atelectasis or total lung collapse. Additionally, mucus retention can impair gas exchange, leading to hypoxemia and hypercapnia [6].

When mucus becomes lodged within the bronchial tree, it can result in segmental, lobar, or complete lung collapse, depending on the location and extent of the obstruction. As the trapped gas is absorbed by surrounding capillaries, and no new air enters the space, the distal lung segment eventually collapses. When the obstruction involves a main bronchus, complete lung collapse can ensue [7].

Recent studies have shown that elderly patients with dementia, especially those with cerebral atrophy or lacunar infarcts, may experience a marked depression of cough reflex sensitivity, putting them at increased risk for aspiration pneumonia and mucus retention. Although the cough reflex starts in the brainstem, research now shows that higher parts of the brain, like the cerebral cortex, also control coughing. In these patients, both the urge to cough and the motor cough response may be blunted, which compromises airway clearance and predisposes them to airway obstruction [8].

Patients with COPD are particularly prone to airway hypersecretion, making mucus plugging more common among smokers. Smoking has been shown to impair mucociliary clearance by reducing ciliary function and increasing mucus viscosity [9]. Cigarette smoke or infection can cause goblet cell overgrowth, reduce ciliated cells, and increase mucus production, leading to airway blockage. This contributes to COPD and worsens patients’ quality of life. Around 50% of COPD patients have chronic mucus hypersecretion (CMH), which triples their risk of death [10].

Chronic disruption of mucociliary clearance can lead to persistent infection, pneumonia, and bronchiectasis, as seen in diseases like cystic fibrosis. This impairment also contributes to increased pulmonary vascular resistance [11]. Conditions such as cystic fibrosis and COPD are characterized by elevated mucin production, which raises the risk of mucus plug formation and airway obstruction [12]. One study demonstrated that cilia alone cannot move fluid upward but can transport mucus, underscoring that the unique biophysical properties of mucus are essential for effective clearance against gravity, an aspect often overlooked in current models [13]. In mouse models, mucus hyperconcentration has been shown to cause mucus adhesion to airway walls, promoting infection and chronic inflammation through mechanisms such as hypoxia, irritant retention, and immune dysregulation. Improving mucus clearance, therefore, holds promise as a therapeutic strategy for chronic mucus-obstructive lung diseases [14].

The perioperative period is another high-risk setting for mucus plugging and atelectasis. General anesthesia induces diaphragmatic paralysis and reduces alveolar distending pressures, contributing to alveolar collapse. Resorptive atelectasis may also occur when gas becomes trapped distal to an obstruction. Additionally, anesthetic agents such as halothane have been shown to disrupt surfactant function. These factors increase the risk of mucus plug formation in postoperative patients with diminished lung capacity, impaired respiratory drive, or reduced ability to cough-conditions commonly seen in individuals with dementia or those recently extubated [15].

During bedside mucus clearance, patient positioning plays a critical role. Gravity can assist in mobilizing secretions toward larger, ciliated airways, promoting more effective clearance. Cilia are better able to transport mucus across non-ciliated regions when aided by gravity, allowing secretions to drain more efficiently from the lungs [16,17].

Preventative measures, called airway clearance techniques, can be taken to prevent obstructions and lung collapse. These techniques use methods like postural drainage, chest percussion, and vibration, and they are vital in diseases with mucus buildup, such as cystic fibrosis, COPD, and neuromuscular disorders. They help break the cycle of infection, inflammation, and long-term lung damage [18].

Nasotracheal suction is a commonly employed bedside technique for clearing airway secretions in non-intubated patients [19]. In contrast, endotracheal or tracheostomy suction is used in patients with secured airways on mechanical ventilation. Blind nasotracheal suction has also been described as a method for obtaining bronchoalveolar lavage specimens in critically ill patients with suspected pneumonia [20].

While routine nasotracheal suctioning is widely performed by nurses, respiratory therapists, and physicians, targeted nasotracheobronchial suction, directed into the main bronchi to remove obstructive mucus plugs, requires specific skill and anatomical knowledge. Our two reported cases demonstrate that this technique can be safely and effectively performed at the bedside in selected patients, offering a viable alternative to bronchoscopy. With the increasing aging population in the US and around the world, patients will be living with conditions with compromised ability to cough and clear airway secretions. Also, patients who choose palliative or comfort care and limit more invasive procedures may need this simple bedside method to clear secretions from the main airways, avoiding the need for sedation, anesthesia, or invasive procedural support.

## Conclusions

Both cases presented in this report demonstrate that blind nasotracheobronchial suctioning successfully cleared mucus plugs and restored ventilation to the collapsed lung. This technique can be safely and effectively performed at the bedside, making it a valuable option for patients in whom invasive procedures such as bronchoscopy are either contraindicated or declined. With proper training, physicians, nurses, respiratory therapists, and other healthcare providers can safely perform nasotracheobronchial suctioning to expand the collapsed lung. 
